# Supplementary material for: A thematic analysis of flu vaccine hesitance in ethnically minoritised communities in Liverpool
Source: PLoS One. 2025 Nov 3;20(11):e0333602. doi: 10.1371/journal.pone.0333602 (PMC12582489; doi:10.1371/journal.pone.0333602)
Supplement: S1 File — (PDF) [file pone.0333602.s001.pdf]

A thematic analysis of flu vaccine hesitance in ethnically minoritised communities in Liverpool

S1 File. Interview and Focus Group Topic Guides for each Participant Group

TOPIC GUIDE FOR PUBLIC PARTICIPANTS

Seasonal influenza vaccine hesitancy in ethnically diverse groups

LJMU's Research Ethics Committee Approval Reference: 23/PSY/081

Date of visit: ________________________ Place: _________________________________

Interviewer: ____

Participant ID: ____

Interviewee(s) Socio-demographic characteristics

Gender: __________________________ Age: ___________________________

Ethnicity: ____________________ Religion: ________________________________

Occupation: ____

Part of Cheshire/Merseyside reside in: ____

Other members of household and their ages (any dependents / caring responsibilities?):

____

____

____

Introduction (by researcher)

- On self
    - Liverpool John Moores University (LJMU) postgraduate student undergoing stage 1 Health Psychology training (DC) or research assistant at LJMU School of Psychology since September (AP)
    - Working with university staff of the psychology department and primary healthcare staff such as GPs in Cheshire and Merseyside on this project
    - Interviewing adults who are eligible for flu vaccines and from ethnically diverse communities in Cheshire and Merseyside
- Aims and purpose of the study
    - Explore experience of the flu, vaccines and reasons for hesitance or uptake, public health messaging and health service access, and how this can be improved
    - Hear your personal experience of flu and COVID vaccines/relevant support or treatment you may have received or, if you haven't, what you think of accessing this
- Confidentiality
    - Your interview and anything you speak about will be confidential: It will not be shared with anyone outside of the study team of LJMU, unless you tell me something that suggests that you might seriously harm yourself or others

- Rights
  - Can stop at any point or take a break
  - Can skip questions if you do not want to answer
- Recording
  - Ok to record the interview?
- Any questions before we start?

Questions for all participants

Informed consent (and vaccine literacy)

1. Please tell me what you know about the flu?
2. What do you feel are the risks of you getting vaccinated or not for the flu?
3. Please tell me about your experience of being offered a flu vaccine *(prompt – who told you about it and what information materials were given)*
4. What did you think about the information you were given?? *(prompt – was it clear? were there words you did not know? Were these explained clearly? Did you need much time to understand the information? Did you/would you need someone to help you understand it?)*
5. How did this compare to information you received about the COVID-19 vaccination?

Vaccine hesitancy/uptake

6. What is your view of vaccination? (e.g., **childhood vaccinations**, COVID-19, BCG (tuberculosis), pet vaccinations) – *(prompt - if express concerns, what are these?)*
7. Have you ever been vaccinated against the flu? What were the reasons behind your decision to get vaccinated or not?
8. Have your opinions changed since the COVID-19 pandemic? If so, how?

8.a. **For unvaccinated participants:** What would help you feel more confident about the flu vaccine?

8.b. **For vaccinated participants:** Will you get the flu vaccine next time it is offered to you? Why/why not?

9. What do you think would happen in the case of a bad reaction that you think was caused by a vaccine? *(prompt – Would you report it? How would you be supported? How would you like to be supported?)*

Health messaging (and vaccine literacy)

10. Where do you go to for health information/ which health information sources do you trust most? Why?
11. Which health information sources do you trust least? Why? *(if e.g., healthcare professionals mentioned – how could this be improved?)*
12. Who have you spoken to about the flu vaccine? *(Prompt - partner, other family members, friends, religious leaders)*
13. What were their opinions, and how did these influence your views?

Access to healthcare

14. How easily can you access the flu vaccine if you decide to get it? *(prompt – travel, childcare, disability).* What would help you to access it?
15. How do you feel about providing your ethnicity to your GP?
16. How can we support the uptake of the flu vaccine in your community? *(Prompt - assurance from religious leaders, more information about the vaccine and influenza, influenza epidemic/perceived higher level of risk to self or household members, support with access)*
17. What format is best to share information with your community? *Eg written letters, texts, posters, leaflets, videos*
18. Is there anything else you'd like to add?

TOPIC GUIDE FOR COMMUNITY CHAMPIONS

Seasonal influenza vaccine hesitancy in ethnically diverse groups

LJMU's Research Ethics Committee Approval Reference: 23/PSY/081

Date of visit: ________________________ Place: _________________________________

Interviewer: ____

Participant ID: ____

Interviewee(s) Socio-demographic characteristics

Gender: __________________________ Age: ___________________________

Ethnicity: ____________________ Religion: ________________________________

Occupation: ____

Part of Cheshire/Merseyside reside in: ____

Introduction

- On self
  - Liverpool John Moores University (LJMU) postgraduate student undergoing stage 1 Health Psychology training (DC) or research assistant at LJMU School of Psychology since September (AP)
  - Working with university staff of the psychology department and primary healthcare staff such as GPs in Cheshire and Merseyside on this project
  - Interviewing community champions who work with adults from ethnically diverse communities in Cheshire and Merseyside who are eligible for seasonal influenza vaccines
- Aims and purpose of the study
  - Explore community champion experience and views of information provision, raising awareness of flu vaccines, health literacy, informed consent, public health messaging, and health service access for seasonal influenza vaccination
- Confidentiality
  - Your interview and anything you speak about will be confidential: It will not be shared with anyone outside of the study team of LJMU, unless you tell me something that suggests that you might seriously harm yourself or others
- Rights
  - Can stop the interview at any point or take a break
  - Can skip questions if you do not want to answer

- Recording
    - Ok to record the interview?
- Any questions before we start?

Questions for all participants

Personal views on vaccination

9. Please tell me about your role *(prompts – daily basis, how long been in role, what is role in relation to vaccinations, how often talk to people about vaccinations, what type of people)*
10. What do you know about seasonal influenza?
11. How serious do you think this is for people from ethnically diverse communities who are eligible for the flu vaccine?
12. What is your general view on routine vaccination programmes? (e.g., childhood vaccinations, COVID-19, tuberculosis, pet vaccinations) – *(prompt - if express concerns, what are these?)*
13. Why do you think people from ethnically diverse communities are less likely to get the seasonal influenza vaccine?
14. How do you think influenza vaccine acceptance may have changed since COVID-19?

Raising awareness of flu vaccines

15. Who do you think is responsible for raising awareness of seasonal influenza vaccination programmes in people from ethnically diverse communities?
16. What does that process look like? How could it be improved?

Culturally appropriate information provision, informed consent (and vaccine literacy)

17. How are eligible people engaged in conversations about seasonal flu vaccines?
18. Does practice differ when administering health information (such as on influenza and vaccines) to people from different ethnic backgrounds? If so, how?
19. What are the barriers for these people when receiving this information? *(prompt – do people from ethnically diverse communities understand the information? Do they know the words used? If not, are these words explained? Do they need much time to understand the information? Do they need someone to help them understand it?)*
20. What would help you to deliver this information to these different groups?

Health messaging (and vaccine literacy)

21. Where do you think people from ethnically diverse communities go to for health information / which health information sources do you think ethnically diverse communities trust most? Why? *(prompt – concerns around halal status of vaccines, including* seasonal influenza vaccine, *cultural information from their country of origin)*
22. Which health information sources do you think ethnically diverse communities trust least? Why? *(if e.g., healthcare professionals mentioned – how could this be improved?)*
23. What do you know about the halal status of seasonal influenza vaccines? How important is this to people in your community? (*note, children's nasal vaccine has porcine gelatine in, adult flu injections do not)*

Access to healthcare

24. In your experience, are people worried about having an adverse event following immunisation, where they may feel that this was caused by the vaccine? Would they report it if this happened? How would they report this? How might you support them in this?

25. Do you feel supported in engaging with vaccine hesitant/sceptical ethnically diverse communities? What would help you feel supported?
26. How do you think patients feel about providing data on ethnicity to their GP? *(prompt – how do they feel this affects their care, and what are the barriers to providing this information)*
27. What are the barriers to childhood vaccinations? Are they the same as those we have spoken about?
28. What do you think would help to support the uptake of seasonal influenza vaccination by ethnically diverse communities? *(Prompt - assurance from religious leaders, more information about the vaccine and influenza, influenza epidemic/perceived higher level of risk to self or household members, support with access)*
29. Is there anything else you'd like to add?
30. Is there anyone else that you recommend we speak to?

INTERVIEW TOPIC GUIDE FOR PRIMARY HEALTHCARE STAFF

Seasonal influenza vaccine hesitancy in ethnically diverse groups ***IRAS ID: 334970***

Date of visit: ________________________ Place: __________________________________

Interviewer: ____

Participant ID: ____

Interviewee(s) Socio-demographic characteristics

Gender: __________________________ Age: ___________________________

Ethnicity: ____________________ Religion: _________________________________

Occupation: ____

Part of Cheshire/Merseyside reside in: ____

Introduction

- On self
  - Research assistant at Liverpool John Moores University (LJMU), School of Psychology since September
  - Working with university staff of the psychology department and primary healthcare staff such as GPs in Cheshire and Merseyside on this project
  - Interviewing healthcare staff who work with adults from ethnically diverse communities in Cheshire and Merseyside who are eligible for seasonal influenza vaccines
- Aims and purpose of the study
  - Explore health care staff experience and views of information provision, raising awareness of flu vaccines, health literacy, informed consent, public health messaging, and health service access for seasonal influenza vaccination

  Confidentiality
  - Your interview and anything you speak about will be confidential: It will not be shared with anyone outside of the study team of LJMU, unless you tell me something that suggests that you might seriously harm yourself or others
- Rights
  - Can stop the interview at any point or take a break
  - Can skip questions if you do not want to answer
- Recording
  - Ok to record the interview?

- Any questions before we start?

Questions for all participants

Personal views on vaccination

31. Please tell me about your role *(prompts – daily basis, how long been in role, what is role in relation to vaccinations, how often providing vaccinations)*
32. What do you know about seasonal influenza?
33. How serious do you think this is for patients from ethnically diverse communities who are eligible for the flu vaccine?
34. What is your general view on routine vaccination programmes? (e.g., childhood vaccinations, COVID-19, tuberculosis, pet vaccinations) – *(prompt - if express concerns, what are these?)*
35. Why do you think people from ethnically diverse communities are less likely to get the seasonal influenza vaccine?
36. How do you think influenza vaccine acceptance may have changed since COVID-19?

Raising awareness of flu vaccines

37. Who do you think is responsible for raising awareness of seasonal influenza vaccination programmes in patients from ethnically diverse communities?
38. What does that process look like? How could it be improved?

Culturally appropriate information provision, informed consent (and vaccine literacy)

39. How are eligible patients engaged in conversations about seasonal flu vaccines?
40. Does practice differ when administering health information (such as on influenza and vaccines) to people from different ethnic backgrounds? If so, how?
41. What are the barriers for these patients when receiving this information? *(prompt – do patients from ethnically diverse communities understand the information? Do they know the words used? If not, are these words explained? Do they need much time to understand the information? Do they need someone to help them understand it?)*
42. What would help you to deliver this information to these different groups?

Health messaging (and vaccine literacy)

43. Where do you think patients from ethnically diverse communities go to for health information / which health information sources do you think ethnically diverse communities trust most? Why? *(prompt – concerns around halal status of vaccines, including* seasonal influenza vaccine, *cultural information from their country of origin)*
44. Which health information sources do you think ethnically diverse communities trust least? Why? *(if e.g., healthcare professionals mentioned – how could this be improved?)*
45. What do you know about the halal status of seasonal influenza vaccines? How important is this to your patients? (*note, children's nasal vaccine has porcine gelatine in, adult flu injections do not)*

Access to healthcare

46. What do you think would happen in the case of an adverse event following immunisation where the individual feels this was caused by the vaccine? Would they report it? How would you support them? How would you like to be supported?
47. Does the possibility of this situation affect your willingness to encourage seasonal influenza vaccination in people who are hesitant?

48. Do you feel supported in engaging with vaccine hesitant/sceptical ethnically diverse communities? What would help you feel supported?
49. How do you think patients feel about providing data on ethnicity to their GP? *(prompt – how do they feel this affects their care, and what are the barriers to providing this information)*
50. What are the barriers to childhood vaccinations? Are they the same as those we have spoken about?
51. What do you think would help to support the uptake of seasonal influenza vaccination by ethnically diverse communities? *(Prompt - assurance from religious leaders, more information about the vaccine and influenza, influenza epidemic/perceived higher level of risk to self or household members, support with access)*
52. Is there anything else you'd like to add?

INTERVIEW TOPIC GUIDE FOR POLICY PROFESSIONALS

Seasonal influenza vaccine hesitancy in ethnically diverse groups

Seasonal influenza vaccine hesitancy in ethnically diverse groups ***IRAS ID: 334970***

Date of visit: ________________________ Place: _________________________________

Interviewer: ____

Participant ID: ____

Interviewee(s) Socio-demographic characteristics

Gender: __________________________ Age: ___________________________

Ethnicity: ____________________ Religion: ________________________________

Occupation: ____

Part of Cheshire/Merseyside reside in: ____

Introduction

- On self
    - Liverpool John Moores University (LJMU) postgraduate student undergoing stage 1 Health Psychology training (DC) or research assistant at LJMU School of Psychology since September (AP)
    - Working with university staff of the psychology department and primary healthcare staff such as GPs in Cheshire and Merseyside on this project
    - Interviewing healthcare staff who work with adults from ethnically diverse communities in Cheshire and Merseyside who are eligible for seasonal influenza vaccines
- Aims and purpose of the study
    - Explore health care staff experience and views of information provision, raising awareness of flu vaccines, health literacy, informed consent, public health messaging, and health service access for seasonal influenza vaccination

Confidentiality
    - Your interview and anything you speak about will be confidential: It will not be shared with anyone outside of the study team of LJMU, unless you tell me something that suggests that you might seriously harm yourself or others
- Rights
    - Can stop the interview at any point or take a break
    - Can skip questions if you do not want to answer

- Recording
    - Ok to record the interview?
- Any questions before we start?

Questions for all participants

Personal views on vaccination

53. Please tell me about your role *(prompts – daily basis, how long been in role, what is role in relation to vaccinations, how often providing vaccinations)*
54. What do you know about seasonal influenza?
55. How serious do you think this is for people from ethnically diverse communities who are eligible for the flu vaccine?
56. What is your general view on routine vaccination programmes? (e.g., childhood vaccinations, COVID-19, tuberculosis, pet vaccinations) – *(prompt - if express concerns, what are these?)*
57. Why do you think people from ethnically diverse communities are less likely to get the seasonal influenza vaccine?
58. How do you think influenza vaccine acceptance may have changed since COVID-19?

Raising awareness of flu vaccines

59. Who do you think is responsible for raising awareness of seasonal influenza vaccination programmes in people from ethnically diverse communities?
60. What does that process look like? How could it be improved?

Culturally appropriate information provision, informed consent (and vaccine literacy)

61. What is your understanding of the process by which eligible people are engaged in conversations about seasonal flu vaccines?
62. Does practice differ when administering health information (such as on influenza and vaccines) to people from different ethnic backgrounds? If so, how?
63. Are you aware of any barriers reported for these people when receiving this information? *(prompt – do people from ethnically diverse communities understand the information? Do they know the words used? If not, are these words explained? Do they need much time to understand the information? Do they need someone to help them understand it?)*
64. What would help staff to deliver this information to these different groups?

Health messaging (and vaccine literacy)

65. Where do you think people from ethnically diverse communities go to for health information / which health information sources do you think ethnically diverse communities trust most? Why? *(prompt – concerns around halal status of vaccines, including* seasonal influenza vaccine, *cultural information from their country of origin)*
66. Which health information sources do you think ethnically diverse communities trust least? Why? *(if e.g., healthcare professionals mentioned – how could this be improved?)*

Access to healthcare

67. How do you incorporate the expertise of the public and healthcare professionals in developing inclusive policies?
68. What are the barriers to developing inclusive vaccination programmes?

69. What do you think would happen in the case of an adverse event following immunisation where the individual feels this was caused by the vaccine? Would they report it? How would people be supported? How would staff like to be supported?
70. Do you feel the possibility of this situation affects staff willingness to encourage seasonal influenza vaccination in people who are hesitant?
71. Do you think staff feel supported in engaging with vaccine hesitant/sceptical ethnically diverse communities? What would help them feel supported?
72. How do you think patients feel about providing data on ethnicity to their GP? *(prompt – how do they feel this affects their care, and what are the barriers to providing this information)*
73. What are the barriers to childhood vaccinations? Are they the same as those we have spoken about?
74. What do you think would help to support the uptake of seasonal influenza vaccination by ethnically diverse communities? *(Prompt - assurance from religious leaders, more information about the vaccine and influenza, influenza epidemic/perceived higher level of risk to self or household members, support with access)*
75. Is there anything else you'd like to add?
